# Deep learning-based postoperative visual acuity prediction in idiopathic epiretinal membrane

**DOI:** 10.1186/s12886-023-03079-w

**Published:** 2023-08-21

**Authors:** Dejia Wen, Zihao Yu, Zhengwei Yang, Chuanzhen Zheng, Xinjun Ren, Yan Shao, Xiaorong Li

**Affiliations:** Tianjin Key Laboratory of Retinal Functions and Diseases, Tianjin Branch of National Clinical Research Center for Ocular Disease, Eye Institute and School of Optometry, Tianjin Medical University Eye Hospital, 251 Fukang Road, Tianjin, 300384 China

**Keywords:** Deep learning, Idiopathic epiretinal membrane, Best-corrected visual acuity, Optical coherence tomography, Artificial intelligence

## Abstract

**Background:**

To develop a deep learning (DL) model based on preoperative optical coherence tomography (OCT) training to automatically predict the 6-month postoperative visual outcomes in patients with idiopathic epiretinal membrane (iERM).

**Methods:**

In this retrospective cohort study, a total of 442 eyes (5304 images in total) were enrolled for the development of the DL and multimodal deep fusion network (MDFN) models. All eyes were randomized into a training dataset with 265 eyes (60.0%), a validation dataset with 89 eyes (20.1%), and an internal testing dataset with the remaining 88 eyes (19.9%). The input variables for prediction consisted of macular OCT images and diverse clinical data. Inception-Resnet-v2 network was utilized to estimate the 6-month postoperative best-corrected visual acuity (BCVA). Concurrently, a regression model was developed using the clinical data and OCT parameters in the training data set for predicting postoperative BCVA. The reliability of the models was subsequently evaluated using the testing dataset.

**Results:**

The prediction DL algorithm exhibited a mean absolute error (MAE) of 0.070 logMAR and root mean square error (RMSE) of 0.11 logMAR in the testing dataset. The DL model demonstrated a robust promising performance with R^2^ = 0.80, notably superior to R^2^ = 0.49 of the regression model. The percentages of BCVA prediction errors within ± 0.20 logMAR amounted to 94.32% in the testing dataset.

**Conclusions:**

The OCT-based DL model demonstrated sensitivity and accuracy in predicting postoperative BCVA in iERM patients. This innovative DL model exhibits substantial potential for integration into surgical planning protocols.

**Supplementary Information:**

The online version contains supplementary material available at 10.1186/s12886-023-03079-w.

## Background

Epiretinal membrane (ERM) is a fibrocellular layer that develops on the inner surface of retina and causes visual symptoms including impaired visual acuity, metamorphopsia, visual field loss, and even diplopia. A systematic review of over 49,000 individuals indicated that older age and female gender were significantly associated with an increased risk of idiopathic ERM (iERM). IERM affects an estimated population of 30 million adults in the United States, ranging in age from 43 to 86 [1], with up to 20%–35% of iERM cases being bilateral [2, 3]. ERM is characterized by fibrocellular proliferation with or without neurosensory retina wrinkling. The etiology of iERM is unknown. However, secondary ERM may result from reactive wound healing and proliferation. The hypothesis that iERM develops as a result of microbreaks in the internal limiting membrane (ILM) caused by posterior vitreous detachment is widely accepted. The microbreaks in the ILM provide an opportunity for the retinal glial cells or possibly retinal pigment epithelium cells to migrate to the inner surface of the retina and proliferate [4, 5].

Surgical intervention in patients with iERM usually depends on the severity of their symptoms. Compared with delayed surgery, earlier vitrectomy surgery for patients with iERM may result in better long-term visual acuity [3, 6]. According to a meta-analysis of 10 studies (1,482 ERM eyes), the distortion improves but fails to completely resolve after fibrocellular membrane peeling. A postoperative effect is that visual acuity improves by two lines or more on average. However, approximately 10%–20% of iERM patients will have unchanged or worse vision after surgery [7].

Optical coherence tomography (OCT) is a highly sensitive and widely used method for making the diagnosis and assessing the prognosis of ERM [8]. With OCT, the ERM can be identified by a hyperreflective layer or, occasionally, an irregular layer on the retina’s inner surface. Some OCT parameters have been identified as predictors of postoperative visual outcomes. The intact inner photoreceptor and ellipsoid zone were found to be associated with better postoperative visual acuity in iERM patients, whereas photoreceptor disruption was shown to be a predictor of poor postoperative visual acuity [9, 10]. With the OCT B-scan, surgeons could assess the fundus status of iERM eyes on an anatomical scale, but it was challenging to directly convert the morphological images to postoperative visual acuity.

Deep neural networks (DNNs) have revolutionized the field of medical image analysis over the recent decade. Deep learning (DL) algorithms have demonstrated higher discriminative powers compared to those of ophthalmologists in diagnosing diabetic retinopathy, age-related macular degeneration, and potential glaucoma [11]. Given the more intricate and variable anatomical characteristics of the fundus, DL may be effective in predicting postoperative best-corrected visual acuity (BCVA) by incorporating the information from these parameters. Based on preoperative macular OCT images and clinical data, this study aimed to create a DL model capable of predicting the BCVA 6 months after vitrectomy and membrane peeling (VMP). In this study, we also attempted to compare the prediction results of DL with that of a stepwise multiple regression model based on the same independent variables used in the AI model and other important factors influencing BCVA improvement, including the duration of symptoms [12], preoperative BCVA [13], inner segment/outer segment (IS/OS) integrity [9, 14, 15], foveolar detachment [16] and central macular thickness (CMT) [17].

## Methods

### Ethics

This study was approved by the Institutional Review Board of the Tianjin Medical University Eye Hospital (TMUEH, No. 2021KY(L)-03). In compliance with the tenets of the Declaration of Helsinki, all private information was removed in advance, and written consent was obtained from the patients.

### Participants and data collection

This study retrospectively collected OCT images from iERM patients in Tianjin Medical University Eye Hospital (Tianjin, China) from July 2016 to December 2021. The patients’ gender, age, preoperative BCVA, duration of symptoms (e.g., progressive visual impairment and metamorphopsia), lens status, and 6-month postoperative BCVA were retrieved from the electronic medical records. The surgical process of VMP is shown in the online Supplementary Method 1. The inclusion criteria were as follows: (1) patients who underwent ophthalmic examination and were diagnosed with iERM, (2) underwent macular OCT scanning before surgery, (3) underwent uneventful VMP surgeries, and (4) had reliable 6-month postoperative BCVA records. The exclusion criteria included eyes with: (1) secondary ERM and concomitant diseases such as diabetic retinopathy, vein or artery occlusion, and glaucoma; (2) corneal opacity or any other ocular disease that might influence visual acuity; and (3) significant cataract prior to surgery or during the 6-month follow-up period. Spectral domain-OCT scanning was performed using Heidelberg Spectral OCT (Heidelberg Engineering, Germany) on all eyes at baseline. The details of the OCT examination and OCT parameter measurements are described in the online Supplementary Method 2.

Finally, 5,304 qualified OCT images were collected from 442 eyes (12 images from each eye). Specifically, 12 B-scans of each eye were seen as a whole, which was paired with patient data (BCVA). Subsequently, the images were randomized into a training dataset (265 eyes, 60.0% of all the eyes) for model development, a validation dataset (89 eyes, 20.1% of all the eyes) for parameter adjustment, and a testing dataset (88 eyes, 19.9% of all the eyes) for internal test and model evaluation.

### Development of the DL model

**Fig. 1:**
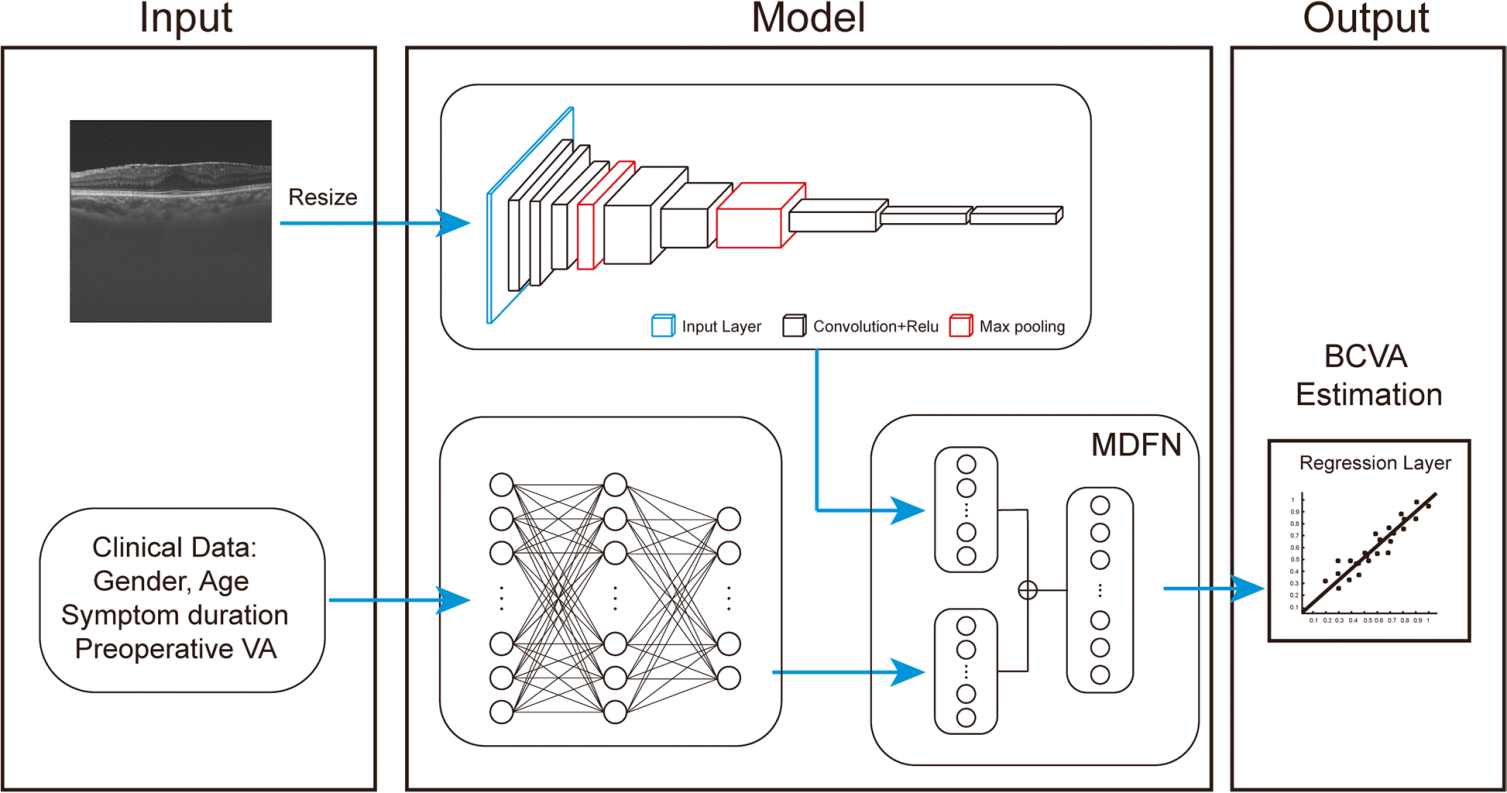
Demonstration of construction of deep learning models. The preoperative b-scan OCT images and clinical data are fed into the model. "CNN" was used to mark the component where the convolutional neural network processes the OCT images, and "MLP" to indicate where the multilayer perceptron handles the structured patient data (including age, pre-operative visual acuity, gender, duration of symptoms, etc.). It eventually outputs the prediction of 6-month postoperative BCVA. BCVA, best-corrected visual acuity; CNN: convolutional neural networks, MLP: multilayer perceptron; MDFN, multimodal deep fusion network

Inception-Resnet-v2 network [1, 18], a DNN with 164 convolutional layers, was employed to estimate the 6-month postoperative BCVA. The parameters of the DNN were initialized against the ImageNet pretrained model. Preoperative OCT images and clinical data were used as the input data. Based on the requirements of Inception-Resnet-v2 network, the OCT images were preprocessed to normalize the input data. We removed saturated pixels from the raw images with an intensity value of 255, and then resized them into 299 × 299 pixels. For each training iteration, a Huber loss was used as the objective loss function, and an adaptive momentum (Adam) algorithm was used to update the network parameters via back-propagation. At every epoch, the performance of network was assessed using the tuning dataset. Back-propagation was repeated for all training images until the network reached satisfactory performance. The network was a regression model output according to the final fully connected layer and a regression layer in the last layer. The batch size was 30, and the learning rate was set to 0.001. The illustration of the pipeline of the work is demonstrated in Fig. 1. In our study, we employ a Convolutional Neural Network (CNN) specifically designed to process the OCT image data. Concurrently, we use a Multilayer Perceptron (MLP) to handle the structured patient data, including age, pre-operative visual acuity, gender, and duration of symptoms. The processed features from both the CNN and MLP are then seamlessly fused to create a comprehensive feature set. Utilizing this enriched feature set, our multimodal deep fusion network is capable of predicting the post-operative visual acuity with improved accuracy. This robust fusion approach capitalizes on the strengths of both image and structured data, thereby enhancing the predictive power of our model.

### Stepwise multiple regression model

The 265 eyes of the training set from the DL model were subsequently used to create a stepwise multiple regression model for the prediction of 6-month postoperative BCVA. Gender, age, preoperative BCVA, symptom duration, preoperative IS/OS integrity, foveolar detachment and CMT were all considered independent variables. Specifically, IS/OS integrity was graded into two groups: intact and disrupted (the hyporeflective disruption of the hyperreflective IS/OS junction) [9, 19]. The coefficient of determination (R^2^) of this stepwise multiple regression model was calculated using the 88 eyes that had been included as the testing dataset in the DL model.

### Evaluation

The metrics used to show the differences between the actual 6-month postoperative BCVA and the estimated BCVA were mean absolute error (MAE), root mean square error (RMSE), and the R^2^, which are defined as:

$${\text{MAE}}=\frac{1}{N}\sum_{i=1}^{N}|{\widetilde{y}}_{i}-{y}_{i}|$$

$${\text{RMSE}}=\sqrt{\frac{1}{N}\sum_{i=1}^{N}({\widetilde{y}}_{i}-{y}_{i}{)}^{2}}$$

where *N* denotes the number of eyes, ${\widetilde{y}}_{i}$ denotes the actual 6-month postoperative BCVA, and ${y}_{i}$ denotes the estimated BCVA.

The prediction error was calculated by subtracting the estimated BCVA from the actual BCVA. The accuracy is defined as the percentage of BCVA prediction errors within ± 0.20 logMAR [20], namely:

$${R}_{e0.2logMAR}=\frac{1}{N}\sum_{i=1}^{N}I(|{\widetilde{y}}_{i}-{y}_{i}|\le 0.2logMAR)$$

where *N*, ${\widetilde{y}}_{i}$, and ${y}_{i}$ have the same meanings as above; the function $I\left(\cdot \right)$ returns 1 if the condition in ($\cdot$) is true, else returns 0.

Gradient-weighted class activation mapping (Grad-CAM), a method for creating "visual explanations", increases the transparency of judgments made by a broad class of DNN-based models. A coarse localization map highlighting the key areas in the image for concept prediction is produced by the Grad-CAM image by using the gradients of any target concept and flowing into the final convolutional layer [21]. To interpret the estimations and increase model transparency, Grad-CAM was used to highlight the discriminative regions of input images in BCVA prediction [21].

### Statistics

Ocular parameters in the training, validation, and testing datasets were compared using an independent *t*-test. The alignment of the estimated and actual BCVA was presented by scatter plots. Bland–Altman plotting was used to visualize the agreement between the estimated and actual values of BCVA. To implement and deploy the network, MATLAB (2020a) was used for training and evaluation. The model was trained on a GPU of NVIDIA RTX3090 with CUDA version 11.3 and cuDNN 8.0. The Inception- ResNet-V2 network architecture used in this work was publicly available in the deep learning tools package. To determine the relationships between the actual and estimated postoperative BCVA values from the DL and stepwise multivariate regression model (gender, age, symptom duration, preoperative BCVA, IS/OS integrity, foveolar detachment and CMT), R^2^ was calculated. Each variable’s entry and exit criteria for the model were determined using the F test, with P values set at 0.05 and 0.1, respectively (in collinearity diagnostic tests, all variance inflation factors were < 10, showing no multicollinearity). The following equation represents a stepwise regression model: y = β0 + β1X1 + β2X2 + ⋯ + βpXp + ε, where β is the regression model’s coefficient, and ε is referred to as the error term. Differences with a P-value of 0.05 or less were considered statistically significant.

## Results

Of the 485 retrospectively enrolled participants, 429 individuals (442 eyes) were eventually included in the current study, while the remaining 56 (11.2%) individuals whose images lacked clinical data or were low-quality OCT images were excluded. The details of these separate datasets and other essential information included in the final analysis are shown in Table 1. No significant differences in gender, age, preoperative BCVA, duration of symptoms, IS/OS integrity, foveolar detachment, CMT, and 6-month postoperative BCVA were identified among the subgroups (*p* > 0.05). All participants were of the same ethnicity (Han Chinese).

**Table 1:** Baseline characteristics of the eyes

Baseline characteristics of iERM eyes	Training set (*n* = 265)	Validation set (*n *= 89)	Testing set (*n* = 88)
Age (years)	66.32 ± 9.13	67.41 ± 7.47	66.23 ± 8.42
Gender, female (%)	58.98%	59.17%	64.28%
Symptom duration (years)	1.19 ± 1.06	1.06 ± 0.79	1.09 ± 0.96
IS/OS integrity (disrupted)	0.38 ± 0.24	0.35 ± 0.19	0.33 ± 0.47
Foveal detachment (%)	9.24%	10.06%	9.48%
CMT(μm)	402.91 ± 63.72	415.36 ± 59.32	411.36 ± 57.44
Pre-op BCVA (log MAR)	0.56 ± 0.31	0.55 ± 0.42	0.55 ± 0.30
Post-op BCVA (log MAR)	0.31 ± 0.24	0.32 ± 0.29	0.32 ± 0.24

*LogMAR* Logarithm of the minimum angle of resolution, *BCVA* Best corrected distance visual acuity, *IS/OS* Inner segment/outer segment, *CMT* Central macular thickness, *Pre-op* Preoperative, *Post-op* postoperative

**Fig. 2:**
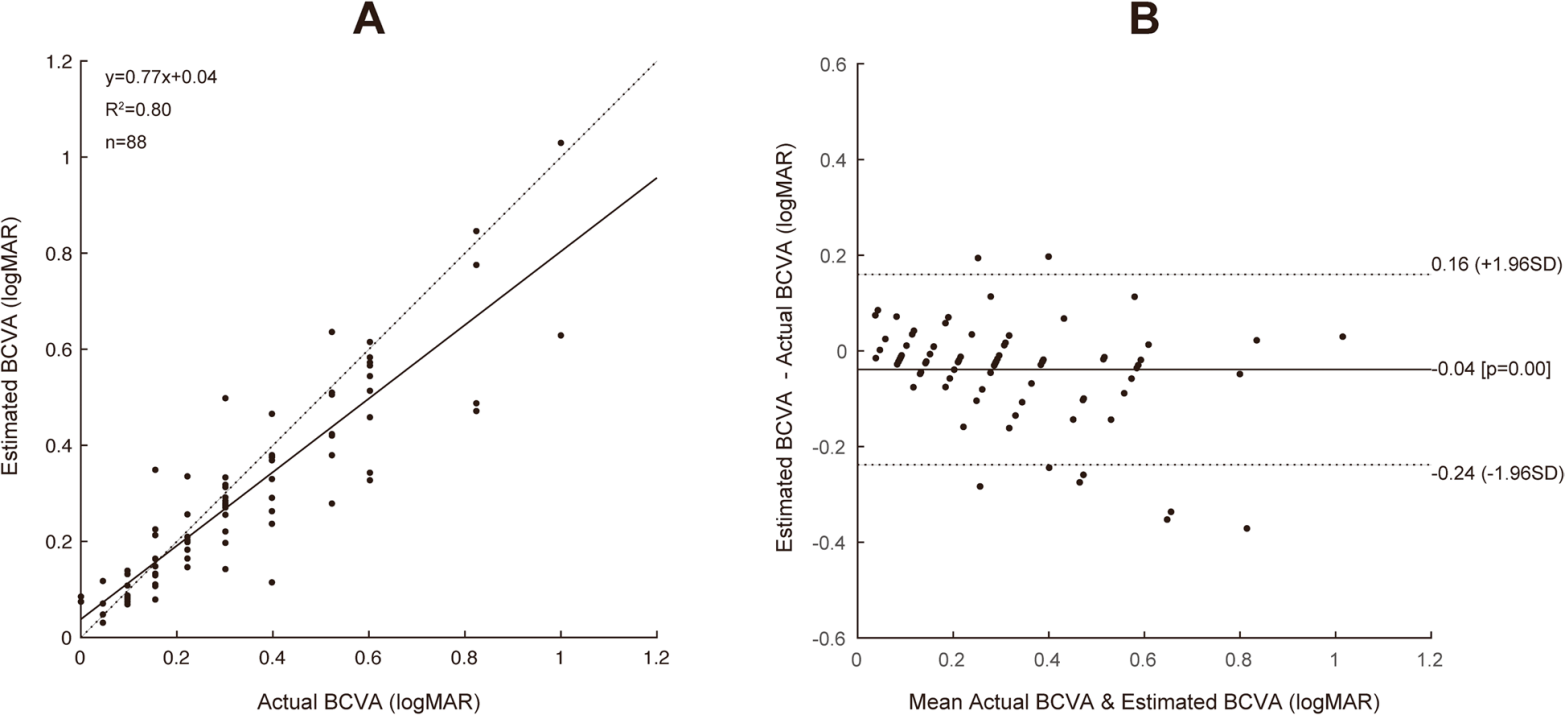
The scatter plots of the predicted BCVA and the actual BCVA in the testing dataset (**A**). The Bland–Altman plots of the predicted BCVA and the actual BCVA in the testing datasets (**B**). The three dotted lines show the mean differences and the 95% confidence level of the difference. BCVA, best-corrected distance visual acuity; logMAR, the logarithm of the minimum angle of resolution

The prediction algorithm demonstrated promising outcomes with MAE of 0.070 logMAR and RMSE of 0.11 logMAR. The estimated and actual values are shown in the scatter plot (Fig. 2A), with the coefficient of determination R^2^ = 0.80. In Bland–Altman plots, the mean difference of BCVA was 0.04 (95% CI, -0.24 to 0.16), with 10.22% (9/88 eyes) measurement points located outside the 95% limits of agreement (Fig. 2B). Based on the testing dataset, the univariate regression model revealed preoperative BCVA and IS/OS integrity were significantly associated with postoperative BCVA (*P* < 0.001, Table 2). According to the stepwise analysis, preoperative BCVA was associated with 6-month postoperative BCVA (β = -0.026, *P* < 0.001). The R^2^ and MAE of the stepwise multiple regression model were 0.49 and 0.12, respectively.

**Table 2:** Linear regression analyses of the associations of postoperative BCVA with baseline characteristics

	Univariate model		Multivariate model	
	β value (95% CI)	*P* value	β value (95% CI)	*P* value
Age (years)	-0.002 (-0.006 to 0.000)	0.065	-	-
Gender (male)	0.060 (-0.049 to 0.061)	0.826	-	-
Symptom duration	0.025 (0.005 to 0.045)	0.013	-	-
IS/OS integrity (disrupted)	-0.13 (-0.184 to -0.075)	< 0.001	-	-
Pre-op BCVA	0.608 (0.545 to 0.671)	< 0.001	-0.002 (0.537 to 0.664)	< 0.001
Foveal detachment	0.023 (-0.091 to 0.137)	0.24	-	-
CMT	0.009 (-0.008 to 0.026)	0.16	-	-

*BCVA* Best corrected distance visual acuity, *IS/OS* Inner segment/outer segment, *CMT* Central macular thickness, *Pre-op* Preoperative, *Post-op* Postoperative

**Fig. 3:**
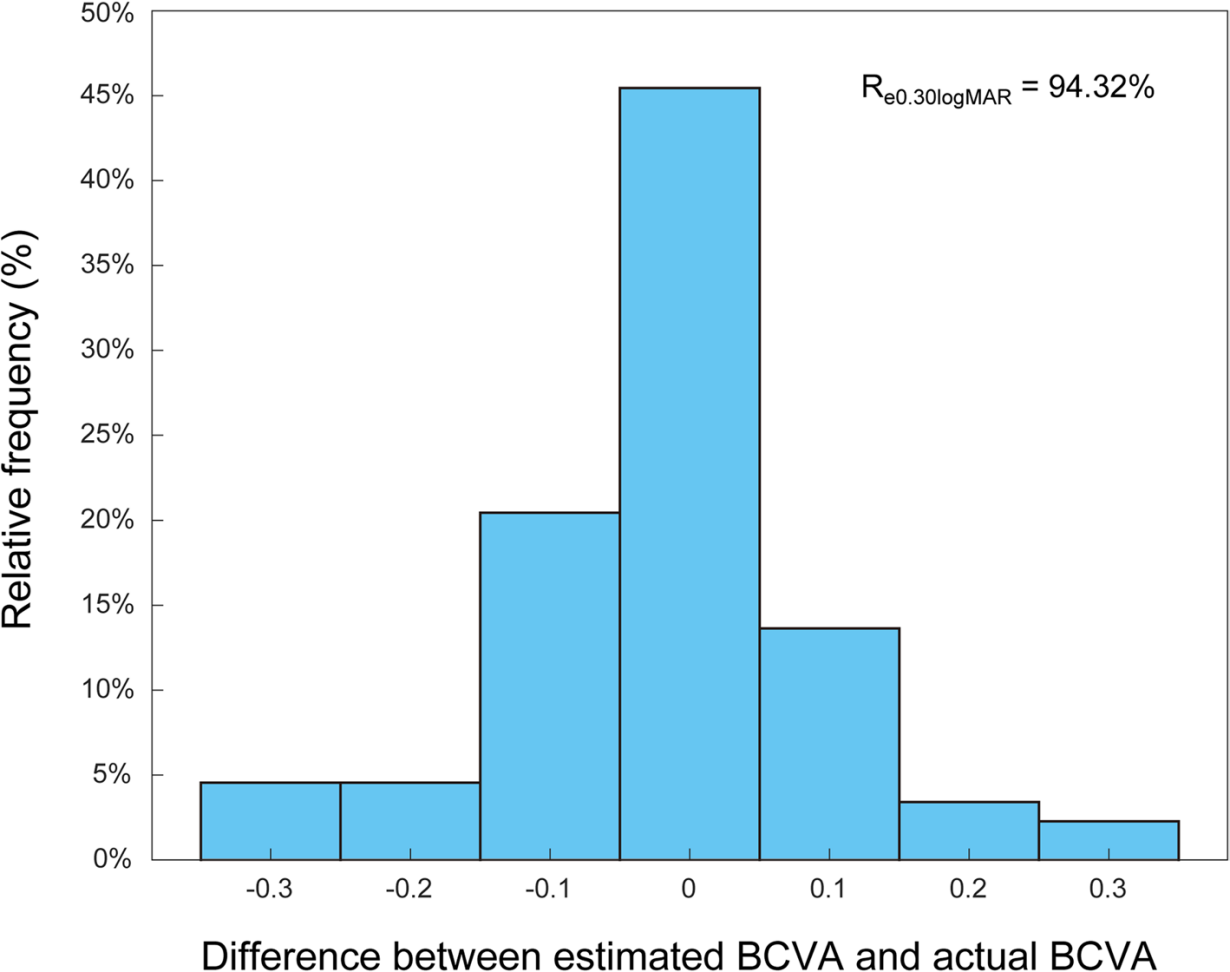
The distribution of the difference between the estimated BCVA and the actual BCVA in the testing dataset. All values are provided in logMAR units. The vertical axis indicates the relative frequency of each BCVA delta value. BCVA, best-corrected visual acuity; logMAR, the logarithm of the minimum angle of resolution; Re0.20logMAR, the percentage of BCVA prediction errors within ± 0.20 logMAR

The distributions of the difference between the actual and the estimated values of BCVA are shown in Fig. 3. The percentages of the prediction errors within ± 0.20 logMAR were 94.32% in the separate testing dataset.

**Fig. 4:**
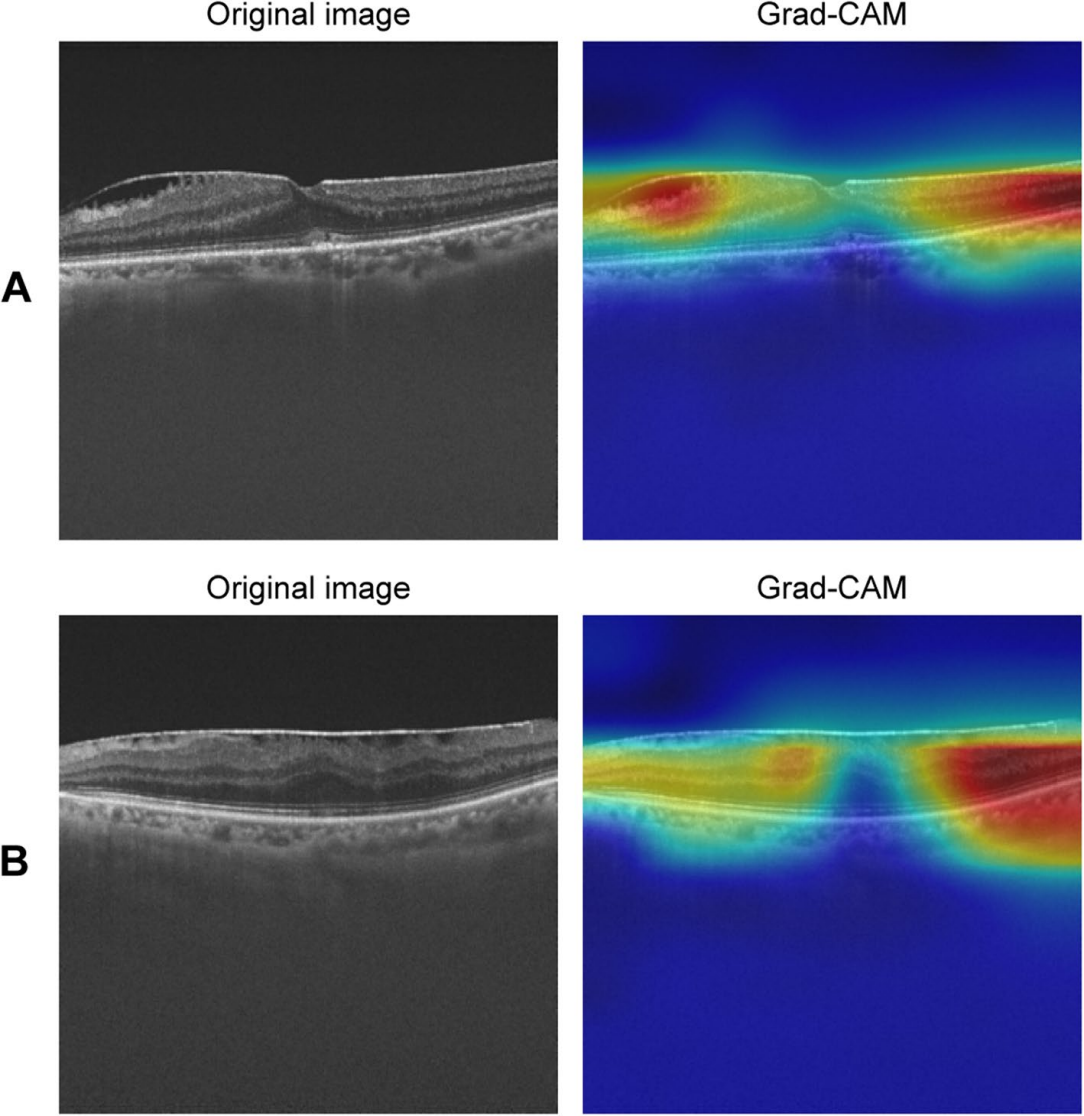
Visualization of the visual acuity recovery prediction. Representative cases of Grad-CAM visualization in the good postoperative vision eyes (**A**). and the poor postoperative vision eyes **(B)**. Grad-CAM, gradient-weighted class activation mapping

To visualize the prediction process, the Grad-CAM presented a highly discriminative region of OCT scanning when predicting the BCVA (Fig. 4). Representative cases of the good postoperative eye (Fig. 4A) and poor vision eye (Fig. 4B) were demonstrated, showing the highly discriminative region of OCT scanning when predicting the BCVA.

## Discussion

Since iERM is inevitably complicated by macular complications, such as macular edema, disorganization of retinal inner layers, and photoreceptor disruption, the visual benefit from VMP remained hard to predict preoperatively. Therefore, we developed a DL model that could predict the post-VMP BCVA of iERM eyes using preoperative macular OCT images and clinical data as input.

Regarding postoperative recovery and rehabilitation, most patients are concerned about postoperative BCVA, which is a scientific indicator of how they are affected in their daily lives. Owing to the satisfactory repeatability and reproducibility of OCT [22], B-scan OCT parameters, such as photoreceptor junction and CMT, have been used as the visual predictors of iERM surgery [23]. A previous study demonstrated choroidal circulation might predict visual outcomes using OCT angiography [21]. Another study investigated the impact of CMT, adherence-zone area, and number of marginal retinal folds on postoperative visual acuity using en-face OCT images [24]. However, these findings on OCT scanning were summarized from only a few parameters and evaluated subjectively [23, 25]. We proposed accurately predicting BCVA using DL algorithms to enable more objective examination and quantitative analysis of the OCT images and clinical data.

In recent years, artificial intelligence (AI) technology, particularly DL, has been widely applied in processing highly complex tasks, such as image segmentation and medical image classification [26]. The convolutional neural network, which generated the postoperative BCVA predictions automatically, consists of multiple convolutional and pooling layers for automatic feature extraction. DL-based AI has been reported to be capable of predicting visual outcomes of retinal or macular diseases, such as neovascular age-related macular degeneration, diabetic macular edema, or retinopathy of prematurity [27, 28]. OCT scanning of macular have been proposed in previous studies to provide millions of morphological parameters associated with BCVA [29]. Although VMP has been proven to be an effective treatment technique for iERM the association between the OCT morphology and the visual outcomes, as assessed 6-month postoperatively, has never been reported. Hence, it is feasible and meaningful to employ DL to predict postoperative BCVA.

In the present study, Grad-CAM helps ophthalmologists determine whether the predictive results are credible by highlighting the decision-making regions. While it effectively visualizes the importance of specific regions, it does not provide a measure of the model's certainty regarding its predictions. The reason why we did not look at the center but looked at the periphery is that it is appropriate to use the deformation of the periphery to measure the load on the neural retina of ERM. Moreover, GRAD-CAM's dependence on gradient information may not always capture all relevant features in an OCT image, as the gradients can be sensitive to minor perturbations and noise, potentially resulting in less accurate heatmaps.

In the present study, we compared the DL model to the stepwise regression model from clinical data, including age, gender, preoperative BCVA, symptom duration, IS/OS integrity, foveolar detachment and CMT. We demonstrated that OCT-based DL model performed better than logistic regression in predicting postoperative BCVA. Despite similar methods, previous investigation differed in the prediction task assigned to the model. The previous investigation focused on binary classification (whether the VA will improve by ≥ 15 letters or not), while our study specifically aimed to predict the postoperative BCVA (the predicted BCVA could be generated automatically) [30]. To our knowledge, this is the first report in literature that directly converts the SD-OCT scans to postoperative BCVA. Despite predicting postoperative BCVA being an intuitively harder task, the promising performance of the OCT-based DL model was demonstrated. Taking the preoperative clinical data and OCT images as input data, our DL-based model has automatically predicted the postoperative BCVA with acceptable accuracies in the VMP-treated iERM eyes. With this model, ophthalmologists only need to input clinical and macular OCT images; then postoperative BCVA would be predicted and generated automatically. Visual acuity prediction could provide valuable guidance for patients to understand the prognosis and make a surgical decision. Accurate prediction of postoperative BCVA can give patients a reasonable expectation and help ophthalmologists deliver appropriate treatment solutions.

It might also help with surgical decision-making, such as choosing more advanced surgical techniques. For visually significant iERM patients, a previous study demonstrated intravitreal injection of 0.7 mg dexamethasone (Ozurdex®) at the conclusion of vitrectomy could significantly decrease macular edema and improve visual outcomes [31]. When the prognosis assessment after vitrectomy and membrane peeling is unfavorable, more advanced surgical techniques, such as intraoperative intravitreal dexamethasone implant [32], can be recommended to patients.

There are several limitations to this study. First, the manually measured macular OCT parameters are prone to measurement errors. Nevertheless, the manual measurements by spectral-domain OCT were proven highly reproducible and repeatable in previous studies [22]. Second, the accuracy of DL algorithms was subject to the quality of OCT images. We ruled out ERM eyes secondary to vitrectomy or with refractive medium opacity. Third, the conclusion of the study is tentative, given the relatively small sample size and short-term follow-up [33]. However, this study offers valuable insights into the predictive ability of the established DL model for patients with iERM. A further prospective multicenter trial is required to assess the accuracy and reliability. In addition, cataract occurs concomitantly with cataract in iERM eyes [34], we have defined the exclusion criteria to reduce the interference of cataract.

## Conclusions

In conclusion, it has been demonstrated that the DL model could precisely predict the post-VMP BCVA based on the preoperative OCT images and clinical data via DL-based AI. The prediction model is expected to assist iERM patients in better understanding the postoperative prognosis and making a reasonable surgical decision.

### Supplementary Information

**Additional file 1: Method 1.**

**Additional file 2: Method 2.**

**Additional file 3: Supplementary figure 1.**

## Data Availability

The data and materials are available upon request from the corresponding author at xiaorli@163.com.

## Abbreviations

DL: Deep learning
OCT: Optical coherence tomography
iERM: Idiopathic epiretinal membrane
BCVA: Best-corrected visual acuity
MAE: Mean absolute error
RMSE: Root mean square error
ERM: Epiretinal membrane
ILM: Internal limiting membrane
DNNs: Deep neural networks
VMP: Vitrectomy and membrane peeling
IS/OS: Inner segment/outer segment
CMT: Central macular thickness
CNN: Convolutional neural networks
MLP: Multilayer perceptron
Grad-CAM: Gradient-weighted class activation mapping
AI: Artificial intelligence
