# Supplementary material for: Deep learning-based postoperative visual acuity prediction in idiopathic epiretinal membrane
Source: BMC Ophthalmol. 2023 Aug 21;23:361. doi: 10.1186/s12886-023-03079-w (PMC10440890; doi:10.1186/s12886-023-03079-w)
Supplement: Supplementary file 1 — Additional file 1: Method 1. [file 12886_2023_3079_MOESM1_ESM.pdf]

Method 1. Surgical Process of VMP

After retrobulbar anesthesia, the VMP surgeries were performed by senior vitreoretinal specialists (Xiaorong Li, Xinjun, Ren, Yan Shao, Juping Liu). Three-port vitrectomy (25-gauge) was performed to remove the core vitreous and shave the peripheral vitreous with caution, avoiding iatrogenic holes. The epiretinal membrane around the macular was gently peeled off circularly. We left transconjunctival incisions self-healed or sutured according to the gas leak. No restrictions were required on patient position after the surgery.
