# Supplementary material for: Deep learning-based postoperative visual acuity prediction in idiopathic epiretinal membrane
Source: BMC Ophthalmol. 2023 Aug 21;23:361. doi: 10.1186/s12886-023-03079-w (PMC10440890; doi:10.1186/s12886-023-03079-w)
Supplement: Supplementary file 2 — Additional file 2: Method 2. [file 12886_2023_3079_MOESM2_ESM.pdf]

Method 2. OCT Examination and OCT Images Collection

In the present study, spectral domain-OCT scanning was performed using Heidelberg Spectral OCT (Heidelberg Engineering, Germany) on all eyes. A 20 x 20 volume acquisition protocol was used by senior technicians to obtain a set of high-speed scans. Following the protocol, 12 horizontal cross-sectional raster scans were obtained. The line spacing of the OCT images was 0.24 mm. We included all OCT images with a quality index of >6. Imaging through the center of the macular was performed based on dynamic observation of the red-free image on the monitor of the OCT machine. All OCT scans were exported in the TIFF format to ensure a consistent image format. One of the authors (Zihao, Yu) collected all OCT scans and matched them with patient data from the electronic medical records.
