# Supplementary material for: Deep learning-based postoperative visual acuity prediction in idiopathic epiretinal membrane
Source: BMC Ophthalmol. 2023 Aug 21;23:361. doi: 10.1186/s12886-023-03079-w (PMC10440890; doi:10.1186/s12886-023-03079-w)

Supplementary Fig. 1 The scatter plots of the pre-operative BCVA and post-operative BCVA in the training, validation and testing dataset **(A)**. The scatter plot of the pre-operative BCVA and post-operative BCVA in the training dataset **(B)**. The scatter plot of the pre-operative BCVA and post-operative BCVA in the validation dataset **(C)**. The scatter plot of the pre-operative BCVA and post-operative BCVA in the testing dataset. BCVA, best-corrected distance visual acuity; logMAR, the logarithm of the minimum angle of resolution.

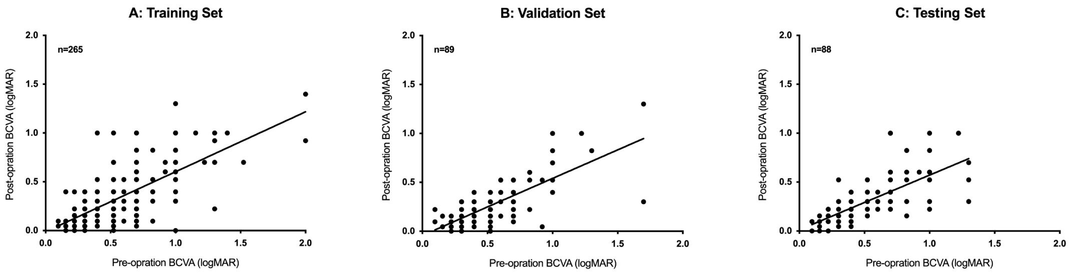

Supplement: Supplementary file 3 — Additional file 3: Supplementary figure 1. [file 12886_2023_3079_MOESM3_ESM.pdf]
